# Musculoskeletal manifestations occur predominantly in patients with later-onset familial Mediterranean fever: Data from a multicenter, prospective national cohort study in Japan

**DOI:** 10.1186/s13075-018-1738-1

**Published:** 2018-11-20

**Authors:** Yushiro Endo, Tomohiro Koga, Midori Ishida, Yuya Fujita, Sosuke Tsuji, Ayuko Takatani, Toshimasa Shimizu, Remi Sumiyoshi, Takashi Igawa, Masataka Umeda, Shoichi Fukui, Ayako Nishino, Shin-ya Kawashiri, Naoki Iwamoto, Kunihiro Ichinose, Mami Tamai, Hideki Nakamura, Tomoki Origuchi, Kazunaga Agematsu, Akihiro Yachie, Junya Masumoto, Kiyoshi Migita, Atsushi Kawakami

**Affiliations:** 1 Department of Immunology and Rheumatology, Unit of Advanced Preventive Medical Sciences, Nagasaki University Graduate School of Biomedical Sciences, 1-7-1 Sakamoto, Nagasaki, 852-8501 Japan; 2 Department of Infection and Host Defense, Graduate School of Medicine, Shinshu University, 3-1-1 Asahi, Matsumoto, 390-8621 Japan; 3 Department of Pediatrics, School of Medicine, Kanazawa University, 13-1 Takaramachi, Kanazawa, 920-8641 Japan; 4 Proteo-Science Center, Ehime University, 3 Bunkyo-cho, Matsuyama, 790-8577 Japan; 5 Department of Rheumatology, Fukushima Medical University School of Medicine, 1 Hikariga-oka, Fukushima, 960-1295 Japan

**Keywords:** Familial Mediterranean fever, *MEFV* gene, Young onset, Late onset, Musculoskeletal manifestations

## Abstract

**Background:**

We showed previously that Japanese individuals with familial Mediterranean fever (FMF) have a more atypical phenotype compared to endemic areas. The clinical differences between young-onset FMF (YOFMF), adult-onset FMF (AOFMF), and late-onset FMF (LOFMF) in Japan are unclear.

**Methods:**

We enrolled 395 consecutive patients. We defined YOFMF, AOFMF, and LOFMF as the onset of FMF at < 20, 20–39, and ≥ 40 years of age, respectively. We compared clinical manifestations and *MEFV* mutations patterns among these groups.

**Results:**

Median ages at onset were YOFMF 12.5 years (*n* = 182), AOFMF 28 years (*n* = 115), and LOFMF 51 years (*n* = 90). A family history, *MEFV* mutations in exon 10, and more than two *MEFV* mutations were significantly more frequent in the earlier-onset groups (*p* < 0.01, *p* < 0.0001, and *p* < 0.001, respectively). In the accompanying manifestations, thoracic and abdominal pain were significantly more frequent in the earlier-onset groups (*p* < 0.01 and *p* < 0.0001, respectively), whereas arthritis and myalgia were significantly more frequent in the later-onset groups (*p* < 0.0001 and *p* < 0.01, respectively). The multiple logistic regression analysis revealed that the presence of *MEFV* exon 10 mutations and earlier onset were significantly associated with serositis, whereas the absence of *MEFV* exon 10 mutations, later onset, and the presence of erysipelas-like erythema were significantly associated with musculoskeletal manifestations. There was no significant between-group difference in the responsiveness to colchicine.

**Conclusions:**

Our results indicate that the later-onset FMF patients had a lower percentage of *MEFV* mutations in exon 10 and predominantly presented arthritis and myalgia. It is important to distinguish their FMF from other inflammatory diseases.

**Electronic supplementary material:**

The online version of this article (10.1186/s13075-018-1738-1) contains supplementary material, which is available to authorized users.

## Background

Familial Mediterranean fever (FMF) is an autoinflammatory disease caused by Mediterranean fever (*MEFV*) gene mutations located on the short arm of chromosome 16 (16 pm 13.3) [1, 2]. FMF is characterized by recurrent and self-limiting fever attacks in a short period accompanied by serositis manifestations including peritonitis and pleuritis, musculoskeletal manifestations including synovitis and myalgia, and skin manifestations including erysipelas-like skin lesions [3–6]. A patient’s FMF can be classified as a typical or an atypical case based on clinical findings and genetic testing [7–9]. According to the Tel Hashomer criteria, a typical case is characterized by fever attacks of ≥ 38.0 °C and lasting 12–72 h accompanied by pleuritis, nonlocalized peritonitis, and monoarthritis of the hip, knee, or ankle [7], whereas an atypical case is characterized by fever attacks of < 38.0 °C, lasting only a short period (i.e., 6–12 h) or lasting a long period (72 h–7 days), abdominal pain without definitive peritonitis, localized peritonitis, or arthritis outside the typical sites (i.e., hip, knee, and ankle) [7].

FMF is most prevalent in individuals in the Mediterranean and Middle Eastern regions, especially in Turks, Arabs, Armenians, and non-Ashkenazi Jews [10, 11]. However, FMF cases have increasingly been reported in some countries outside these regions, such as Japan and the USA [12]. In particular, Japanese FMF cases with *MEFV* mutations were described for the first time in 2002 [13], and there is accumulating evidence showing the characteristics of FMF in Japan [9, 14–19]. The frequency of FMF cases with high-penetrance *MEFV* mutations such as exon 10 is lower in Japan than in Western countries, and FMF cases in Japan have been reported to more often be adult onset and to more often show atypical clinical symptoms [9]. Because of the misunderstanding that FMF is rare in Japan, or that there is a higher percentage of earlier onset in Japan, it is possible that the condition’s diagnosis has been delayed [16].

The onset of FMF in an individual over 40 years of age has been considered rare. A survey of 470 cases showed that approximately 60% of the patients experienced the first attack before 10 years of age, 90% of those experienced the first attack before 20 years of age, and most of the rest of the patients experienced the first attack before 40 years of age [5]. Although there is no definition to classify later-onset FMF including adult-onset FMF (AOFMF) and late-onset FMF (LOFMF), previous studies defined AOFMF and LOFMF as the onset of FMF over 20 and 40 years of age, respectively [20, 21], and revealed that a subgroup of patients with AOFMF or LOFMF is characterized by different demographic, clinical, and probably genetic features [20–23]. However, characteristics of later-onset FMF have not yet been fully elucidated in Japan.

In the present study, using data from a nationwide, multicenter, prospective study in Japan, we compared the clinical characteristics and the distribution of *MEFV* mutations among AOFMF, LOFMF, and YOFMF patients and determined the factors that can distinguish these three groups.

## Methods

### Patients

This was a prospective cohort study registered with the University Hospital Medical Information Network Clinical Trials Registry (#UMIN000015881; http://www.umin.ac.jp/ctr/). The study population consisted of 395 Japanese patients with FMF who were recruited consecutively and prospectively between January 2009 and March 2017 from 106 related centers of Nagasaki University, Shinshu University, Kanazawa University, and Nagasaki Medical Center in Japan. Each of the FMF patients fulfilled the Tel Hashomer criteria [7]. All patients underwent a clinical assessment and provided a blood sample for *MEFV* mutation analyses.

On the basis of the Tel Hashomer criteria, we divided the study patients into two groups: those with typical FMF and those with atypical FMF. The typical FMF patients had suffered typical episodes of peritonitis, pleuritis, monoarthritis, or fever alone as specified in the Tel Hashomer criteria. The atypical FMF patients had suffered an “incomplete” attack. An attack was considered incomplete if it differed from the definition of a typical attack in only one or two of the four following features: temperature < 38 °C; attack duration longer or shorter than specified periods (12 h–3 days), but not shorter than 6 h or longer than 1 week; no signs of peritonitis during an abdominal attack, or signs were localized; and atypical distribution of arthritis.

We defined YOFMF, AOFMF, and LOFMF as the onset of FMF at < 20, 20–39, and ≥ 40 years of age, respectively. We compared clinical manifestations including the characteristics of febrile episodes (duration and frequency), presence of serositis (chest or abdominal pain), arthritis, myalgia, erysipelas-like rash, and response to colchicine. We also compared the three groups’ laboratory findings obtained during an attack including white blood cell count (WBC), C-reactive protein (CRP) level, serum amyloid A (SAA) level, erythrocyte sedimentation (ESR), and IgD level. All patients gave their signed informed consent to be subjected to the protocol, which was approved by the Institutional Review Board of Nagasaki University and related centers (Approval No. 14092946).

### Mutational analysis

We extracted genomic DNA using the Promega Wizard® Genomic DNA Purification Kit (Promega, Madison, WI, USA). We subsequently performed polymerase chain reaction (PCR) using the forward and reverse primers for each exon of the *MEFV* gene as described [9]. We purified PCR products with the reagent ExoSAP-IT™ (GE Healthcare Japan, Tokyo) and sequenced directly, using specific primers and BigDye Terminator v1.1 (Applied Biosystems, Tokyo, Japan).

### Statistical analysis

The demographic, clinical, and genomic characteristics among the YOFMF, AOFMF and LOFMF patients were compared with Fisher’s exact test for discrete variables, and with Wilcoxon’s test for continuous variables. The Kruskal–Wallis test followed by Dunn’s multiple comparisons test were used to compare the groups.

We performed a sensitivity analysis by removing patients with mutations in exon 10 or overlapping rheumatic disease, because mutations of exon 10 in the *MEFV* gene are associated with typical FMF [9] and overlapping rheumatic disease influences clinical symptoms. To determine the independent factors of the patients’ serositis or musculoskeletal manifestations, we performed a multiple logistic regression analysis. We selected variables with *p* < 0.05 by univariate analyses as model 1 (continuous variables for age at onset) or model 2 (binary variables for age at onset). Statistical analyses were performed in JMP pro 13.0 software (SAS Institute, Cary, NC, USA). All reported *p* values are two-sided. *p* < 0.05 was considered significant.

## Results

### Patient characteristics, classification, and complications

A total of 395 patients were enrolled in the study. We excluded eight patients from the analyses due to a lack of age data. The demographic characteristics of the patients with YOFMF (*n* = 182), AOFMF (*n* = 115), and LOFMF (*n* = 90) are presented in Table 1. The median age at diagnosis in the YOFMF, AOFMF, and LOFMF groups was 19, 34, and 58 years, respectively. The groups’ median period between the onset of symptoms and the disease diagnosis was 7, 4, and 2 years, respectively (YOFMF vs others, *p* < 0.0001; others vs LOFMF, *p* < 0.0001). There was no significant difference in gender among the groups. The family history suggestive of FMF was observed in 28%, 17%, and 12% of the YOFMF, AOFMF, and LOFMF groups, respectively, and a significantly higher frequency of family history was observed among the earlier-onset groups (YOFMF vs others, *p* < 0.01; others vs LOFMF, *p* < 0.05).

**Table 1:** Patient characteristics, classification, and complications in patients with YOFMF, AOFMF, and LOFMF (univariate analyses)

Variable	YOFMF (*n* = 182)	AOFMF (*n* = 115)	LOFMF (*n* = 90)	*p* value		
				YOFMF vs AOFMF vs LOFMF	YOFMF vs others	Others vs LOFMF
Age at onset (years)	12.5 (6–15, *n* = 182)	28 (22–33, *n* = 115)	51 (45–61, *n* = 90)	NA	NA	NA
Age at diagnosis (years)	19 (12–30, *n* = 182)	34 (29–39, *n* =115)	58 (48.5–68, *n* = 90)	NA	NA	NA
Interval between disease onset and diagnosis (years)	7 (2–15.5, *n* = 182)	4 (1–11, *n* = 115)	2 (0.5–8, *n* = 90)	< 0.0001	< 0.0001	< 0.0001
Male gender	67/181 (37%)	53/115 (46%)	37/88 (44%)	0.29	0.15	0.81
Family history	51/182 (28%)	20/115 (17%)	11/90 (12%)	0.0055	0.0026	0.018
Typical FMF	111/182 (61%)	57/115 (50%)	46/90 (51%)	0.10	0.041	0.40
AA amyloidosis	2/182 (1%)	5/115 (4%)	3/90 (3%)	0.20	0.11	0.70
Autoimmune or autoinflammatory diseases	9/182 (5%)	12/115(10%)	22/90 (24%)	< 0.0001	0.0003	< 0.0001
RA	0/182 (0%)	0/115 (0%)	9/90 (10%)	< 0.0001	0.0040	< 0.0001
SLE	1/182 (1%)	1/115 (1%)	2/90 (2%)	0.43	0.63	0.23
SS	1/182 (1%)	3/115 (3%)	0/90 (0%)	0.13	0.63	0.58
PM or DM	0/182 (0%)	2/115 (2%)	1/90 (1%)	0.23	0.25	0.55
BD	0/182 (0%)	3/115 (3%)	1/90 (1%)	0.10	0.13	1.00
AOSD	0/182 (0%)	2/115 (2%)	2/90 (2%)	0.16	0.13	0.23
Others (i.e., RS3PE syndrome, Basedow disease, UC, ITP, MS, PBC, and unknown)	7/182 (4%)	1/115 (1%)	7/90 (8%)	0.040	0.98	0.054

Median (interquartile range, number) or number (percentage) presented. *p* values established using Fisher’s exact test or Mann–Whitney *U* test

*YOFMF* young-onset FMF, *AOFMF* adult-onset FMF, *LOFMF* late-onset FMF, *FMF* familial Mediterranean fever, *NA* not available, *AA* amyloid A, *RA* rheumatoid arthritis, *SLE* systemic lupus erythematosus, *SS* Sjögren’s syndrome, *PM* polymyositis, *DM* dermatomyositis, *BD* Behçet’s disease, *AOSD* adult-onset Still’s disease, *RS3PE* remitting seronegative symmetrical synovitis with pitting edema, *UC* ulcerative colitis, *ITP* idiopathic thrombocytopenic purpura, *MS* multiple sclerosis, *PBC* primary biliary cholangitis

The numbers of patients with the typical FMF phenotype according to the Tel Hashomer criteria [7] in the YOFMF, AOFMF, and LOFMF groups were 111 (61%), 57 (50%), and 46 (51%), respectively. We found that the YOFMF group had a significantly higher percentage of patients with typical FMF (YOFMF vs others, *p* < 0.05; others vs LOFMF, *p* = 0.40). In the YOFMF, AOFMF, and LOFMF groups, the rates of amyloidosis as a complication were 1%, 4%, and 3% and the rates of the complication of autoimmune or autoinflammatory diseases were 5%, 10%, and 24%, respectively. Autoimmune or autoinflammatory diseases mainly included diseases such as rheumatoid arthritis (RA), systemic lupus erythematosus (SLE), Sjögren’s syndrome (SS), Behçet’s disease (BD), and adult-onset Still’s disease (AOSD). There was a significantly higher percentage of overlapping autoimmune or autoinflammatory diseases among the later-onset group compared to the other groups (YOFMF vs others, *p* < 0.001; others vs LOFMF, *p* < 0.0001).

### Clinical and laboratory characteristics

The demographic clinical characteristics of the patients with YOFMF, AOFMF, and LOFMF are presented in Table 2. No significant differences were found in the attack frequency or the attack duration among the three groups. Although we found no significant differences in the presence of fever, pericarditis, headache, or erysipelas erythema during the attack, the serositis symptoms such as chest pain and abdominal pain were significantly fewer in the later-onset group: YOFMF vs others, *p* < 0.01; others vs LOFMF, *p* < 0.01; YOFMF vs others, *p* = 0.0001; and others vs LOFMF, *p* < 0.0001. In contrast, musculoskeletal symptoms such as joint pain and myalgia were significantly more frequent in the later-onset group: YOFMF vs others, *p* < 0.0001; others vs LOFMF, *p* < 0.0001; YOFMF vs others, *p* < 0.01; and others vs LOFMF, *p* < 0.01. We examined the efficacy of colchicine therapy in each group and found no significant difference in this parameter among the three groups.

**Table 2:** Clinical and laboratory characteristics in patients with YOFMF, AOFMF, and LOFMF (univariate analyses)

Variable	YOFMF (*n* = 182)	AOFMF (*n* = 115)	LOFMF (*n* = 90)	*p* value		
				YOFMF vs AOFMF vs LOFMF	YOFMF vs others	Others vs LOFMF
Frequency of febrile attack (/month)^a^	1.0 (0.3–1.0, *n* = 157)	1.0 (0.4–1.0, *n* = 97)	0.5 (0.4–1.0, *n* = 74)	0.90	0.66	0.66
Duration of fever attack (days)^a^	2.5 (2–4, *n* = 165)	3 (2–4.8, *n* = 101)	2.5 (2–5, *n* = 71)	0.25	0.25	0.75
Headache	25/161 (16%)	18/91 (20%)	10/65 (15%)	0.65	0.65	0.85
Thoracic pain	82/182 (45%)	38/115 (33%)	22/90 (24%)	0.0025	0.0015	0.0060
Abdominal pain	117/182 (64%)	64/115 (56%)	27/90 (30%)	< 0.0001	0.0001	< 0.0001
Pericarditis	3/161 (2%)	3/91 (3%)	4/65 (6%)	0.25	0.21	0.13
Arthritis	58/182 (32%)	55/115 (48%)	56/90 (62%)	< 0.0001	< 0.0001	< 0.0001
Myalgia	13/161 (8%)	16/91 (18%)	17/65 (26%)	0.0014	0.0012	0.0051
Erysipelas-like erythema	18/182 (10%)	16/115 (14%)	17/90 (19%)	0.11	0.10	0.076
Good response to colchicine	124/126 (98%)	85/87 (98%)	69/71 (97%)	0.84	0.70	0.64
WBC (× 10^3^/μl)^a^	10 (7.2–13, *n* = 126)	9.1 (7.2–12.5, *n* = 64)	9.1 (7.4–12, *n* = 48)	0.84	0.65	0.94
CRP (mg/dl)^a^	7.1 (4.0–12.7, *n* = 132)	7.1 (2.1–11.1, *n* = 65)	9.4 (3.4–15, *n* = 50)	0.12	0.46	0.22
SAA (μg/ml)^a^	85 (206–1246, *n* = 43)	423 (55–1100, *n* = 13)	300 (105–702, *n* = 9)	0.23	0.10	0.18
ESR (mm/h)^a^	40 (24–51, *n* = 39)	41.5 (15.5–56, *n* = 10)	56 (35–90, *n* = 15)	0.063	0.075	0.019
IgD (mg/dl)^a^	4.2 (1.1–10.9, *n* = 17)	1.5 (1.0–2.3, *n* = 3)	10 (1.9–18, *n* = 2)	0.41	0.64	0.46

Median (interquartile range, number) or number (percentage) presented. *p* values established using Fisher’s exact test or Mann–Whitney *U* test

*YOFMF* young-onset FMF, *AOFMF* adult-onset FMF, *LOFMF* late-onset FMF, *FMF* familial Mediterranean fever, *WBC* white blood cell count, *CRP* C-reactive protein, *SAA* serum amyloid A, *ESR* erythrocyte sedimentation, *IgD* immunoglobulin D

We next evaluated laboratory characteristics and found that there were no significant between-group differences in WBC count, CRP level, SAA level, or IgD level during the attack. The median ESR values during the attack in the YOFMF, AOFMF, and LOFMF groups were 40, 41.5, and 56 mm/h, respectively (YOFMF vs others, *p* = 0.07; others vs LOFMF, *p* < 0.05). It is likely that this difference in ESR can be explained by the influence of age.

### Mutational analysis

The results of our demographic mutational analysis of the YOFMF, AOFMF, and LOFMF groups are presented in Table 3. Mutations accompanied by amino acid substitutions of the *MEFV* gene in the YOFMF, AOFMF, and LOFMF patients were observed in 94%, 92%, and 87%, respectively (data not significant). When we compared mutations in the groups by the site of mutations, we found no significant difference in exon 2 or exon 3 in each group. In contrast, mutations in exon 1 in the YOFMF, AOFMF, and LOFMF patients were observed at 6%, 5%, and 0%, respectively (YOFMF vs others, *p* = 0.15; others vs LOFMF, *p* < 0.0001), and mutations in exon 10 were observed in 49%, 30%, and 18%, respectively (YOFMF vs others, *p* < 0.0001; others vs LOFMF, *p* < 0.0001).

**Table 3:** Comparison of mutational analysis in patients with YOFMF, AOFMF, and LOFMF (univariate analyses)

Variable	YOFMF (*n* = 182)	AOFMF (*n* = 115)	LOFMF (*n* = 90)	*p* value		
				YOFMF vs AOFMF vs LOFMF	YOFMF vs others	Others vs LOFMF
*MEFV* mutations (+)	171/182 (94%)	106/115 (92%)	78/90 (87%)	0.12	0.14	0.078
Exon 1 mutations (+)	11/182 (6%)	6/115 (5%)	0/90 (0%)	0.064	0.15	0.016
Exon 2 mutations (+)	127/182 (70%)	83/115 (72%)	61/90 (68%)	0.79	1.00	0.60
Exon 3 mutations (+)	22/182 (12%)	14/115 (12%)	15/90 (17%)	0.54	0.65	0.29
Exon 10 mutations (+)	89/182 (49%)	35/115 (30%)	16/90 (18%)	< 0.0001	< 0.0001	< 0.0001
Heterozygote mutations (+)	22/182 (12%)	13/115 (11%)	5/90 (6%)	0.23	0.32	0.11
More than two *MEFV* mutations (+)	125/182 (69%)	65/115 (57%)	41/90 (46%)	0.0009	0.0009	0.0022

Number (percentage) presented*. p* values established using Fisher’s exact test or Mann–Whitney *U* test

*YOFMF* young-onset FMF, *AOFMF* adult-onset FMF, *LOFMF* late-onset FMF, *FMF* familial Mediterranean fever, *MEFV* Mediterranean fever gene

We analyzed the percentage of patients with two or more *MEFV* mutations among the three groups and found that the rate of having two or more mutations in the *MEFV* gene was higher in the earlier-onset groups: YOFMF vs others, *p* < 0.001; and others vs LOFMF, *p* < 0.01.

### Sensitivity analysis

To conduct a sensitivity analysis, we excluded patients with exon 10 mutation in the *MEFV* gene and patients with rheumatic disease, and reanalyzed the clinical and genetic characteristics among the YOFMF, AOFMF and LOFMF patients. A flow chart of this sensitivity analysis is shown in Additional file 1: Figure S1. As presented in Table 4, we found no significant between-group differences in the proportion of typical FMF, a positive family history, two or more mutations, or chest pain.

**Table 4:** Characteristics of the YOFMF, AOFMF, and LOFMF patients in a sensitivity analysis removing patients with mutations in exon 10 or overlapping rheumatic diseases (univariate analyses)

Variable	YOFMF (*n* = 91)	AOFMF (n = 74)	LOFMF (*n* = 61)	*p* value		
				YOFMF vs AOFMF vs LOFMF	YOFMF vs others	Others vs LOFMF
Typical FMF	33/91 (36%)	25/74 (34%)	25/61 (41%)	0.68	1.00	0.44
Family history	15/91 (16%)	9/74 (12%)	6/61 (10%)	0.47	0.32	0.51
Exon 1 mutations (+)	11/91 (12%)	6/74 (8%)	0/61 (0%)	0.021	0.041	0.0078
Exon 2 mutations (+)	62/91 (68%)	58/74 (78%)	40/61 (66%)	0.20	0.55	0.32
Exon 3 mutations (+)	22/91 (24%)	14/74 (19%)	12/61 (20%)	0.67	0.41	0.86
More than two *MEFV* mutations (+)	48/91 (53%)	39/74 (53%)	24/61 (39%)	0.20	0.42	0.10
Thoracic pain	17/91 (19%)	19/74 (26%)	17/61 (28%)	0.36	0.20	0.38
Abdominal pain	55/91 (60%)	34/74 (46%)	13/61 (21%)	< 0.0001	0.0002	< 0.0001
Arthritis	40/91 (44%)	39/74 (53%)	39/61 (64%)	0.053	0.043	0.036
Myalgia	4/76 (5%)	12/56 (21%)	12/42 (29%)	0.0018	0.0007	0.016

Number (percentage) presented. *p* values established using Fisher’s exact test or Mann–Whitney *U* test

*YOFMF* young-onset FMF, *AOFMF* adult-onset FMF, *LOFMF* late-onset FMF, *FMF* familial Mediterranean fever, *MEFV* Mediterranean fever gene

Interestingly, the presence of mutation in exon 1 and abdominal pain during the attack were significantly more frequently observed in the earlier-onset group: YOFMF vs others, *p* < 0.05; others vs LOFMF, *p* < 0.01; YOFMF vs others, *p* < 0.001; and others vs LOFMF, *p* < 0.0001. In contrast, the later-onset group presented musculoskeletal manifestations more frequently than the earlier-onset group: YOFMF vs others, *p* < 0.05; others vs LOFMF, *p* < 0.05; YOFMF vs others, *p* < 0.001; and others vs LOFMF, *p* < 0.05.

### Identification of independent factors associated with serositis manifestations

To determine which variables are associated with serositis manifestations among the three groups, we evaluated the 20 variables presented in Tables 1, 2, 3. We found that the following nine variables were significantly associated with serositis in the univariate analyses: age at onset, family history, typical FMF, duration of fever attack, arthritis, erysipelas-like erythema, autoimmune or autoinflammatory diseases, exon 3 mutation, and exon 10 mutation.

We selected these variables for a logistic regression analysis and identified three independent factors associated with serositis manifestations: age at onset (odds ratio (OR) 0.43, 95% confidence interval (CI) 0.24–0.78, *p* = 0.0051), autoimmune or autoinflammatory diseases (OR 0.21, 95% CI 0.09–0.55, *p* = 0.0006), and positive exon 10 mutations (OR 10.7, 95% CI 4.15–27.7, *p* < 0.0001) (model 2 in Table 5). Taking these results together, we determined that age at FMF onset, complication of autoimmune or autoinflammatory diseases, and exon 10 mutation in the *MEFV* gene are independent factors that are associated with serositis manifestation.

**Table 5:** Comparison of selected variables for thoracic or abdominal pain in multiple logistic regression analysis (continuous and binary variables)

Variable	*p* value	OR (95% CI)
Model 1		
Age at onset	0.0081	0.979 (0.963–0.995)
Family history	0.4012	1.415 (0.624–3.209)
Typical FMF	0.0437	2.023 (1.018–4.021)
Duration of fever attack	0.7985	1.012 (0.924–1.108)
Arthritis	0.8003	0.926 (0.510–1.681)
Erysipelas-like erythema	0.2824	0.655 (0.302–1.418)
Autoimmune or autoinflammatory diseases	0.0007	0.218 (0.0858–0.557)
Exon 3 mutations (+)	0.7587	0.890 (4.018–26.716)
Exon 10 mutations (+)	< 0.0001	10.361 (4.018–26.716)
Model 2		
Age at onset ≥20 years	0.0051	0.431 (0.238–0.782)
Family history	0.3733	1.445 (0.636–3.284)
Typical FMF	0.0527	1.970 (0.991–3.920)
Duration of fever attack	0.8415	1.009 (0.920–1.107)
Arthritis	0.7936	0.924 (0.509–1.675)
Erysipelas-like erythema	0.1723	0.589 (0.275–1.262)
Autoimmune or autoinflammatory diseases	0.0006	0.216 (0.085–0.545)
Exon 3 mutations (+)	0.7174	0.872 (0.414–1.835)
Exon 10 mutations (+)	< 0.0001	10.718 (4.148–27.694)

Odds ratio (OR), 95% confidence interval (CI), and *p* value in model 1 or model 2 presented

*FMF* familial Mediterranean fever

### Identification of independent factors associated with musculoskeletal manifestations

We next sought to determine variables that are associated with musculoskeletal manifestations among the three groups. We found that the following six variables were significantly associated with musculoskeletal manifestations in the univariate analyses: age at onset, typical FMF, abdominal pain, erysipelas-like erythema, autoimmune or autoinflammatory diseases, and exon 10 mutation. By performing a logistic regression analysis, we identified three independent factors associated with musculoskeletal manifestations: age at onset (OR 1.84, 95% CI 1.12–2.99, *p* = 0.0077), erysipelas-like erythema (OR 4.30, 95% CI 2.04–9.05, *p* < 0.0001), and positive exon 10 mutations (OR 0.54, 95% CI 0.31–0.93, *p* < 0.0027) (model 2 in Table 6). Collectively, these results led us to conclude that age at FMF onset, erysipelas-like erythema, and exon 10 mutation in the *MEFV* gene are independent factors associated with musculoskeletal manifestation.

**Table 6:** Comparison of selected variables for arthritis or myalgia in multiple logistic regression analysis (continuous and binary variables)

Variable	*p* value	OR (95% CI)
Model 1		
Age at onset	0.0006	0.978 (0.965–0.991)
Typical FMF	0.8131	0.940 (0.565–1.564)
Abdominal pain	0.4751	0.842 (0.526–1.348)
Erysipelas-like erythema	< 0.0001	4.153 (1.960–8.802)
Autoimmune or autoinflammatory diseases	0.1988	1.609 (0.773–3.349)
Exon 10 mutations (+)	0.0315	0.550 (0.318–0.950)
Model 2		
Age at onset ≥ 20 years	0.0077	1.840 (1.175–2.882)
Typical FMF	0.7933	0.935 (0.564–1.549)
Abdominal pain	0.2795	0.7745 (0.488–1.229)
Erysipelas-like erythema	< 0.0001	4.295 (2.039–9.050)
Autoimmune or autoinflammatory diseases	0.1224	1.752 (0.851–3.605)
Exon 10 mutations (+)	0.0272	0.541 (0.313–0.935)

Odds ratio (OR), 95% confidence interval (CI), and *p* value in model 1 or model 2 presented

*FMF* familial Mediterranean fever

## Discussion

Our findings clarified the differences in later-onset FMF including AOFMF and LOFMF. Our data showed that later-onset FMF patients have a shorter diagnostic delay, a lower frequency of family history, a lower frequency of typical cases, a higher frequency of complications of autoimmune or autoinflammatory diseases, and a lower frequency of *MEFV* mutations in exons 1 and 10. Importantly, our analyses revealed that later-onset FMF patients predominantly present musculoskeletal manifestations, which is independent of overlapping rheumatic diseases and the *MEFV* mutation in exon 10.

The manifestations of FMF are attributed mainly to the difference in the mutational pattern in the *MEFV* gene [3, 9, 18, 19]. FMF patients with low-penetrance mutations tend to present with milder disease phenotypes and to be diagnosed with atypical FMF [24–26]. In addition, the M694 V mutations in exon 10 mutations, which is high penetrance, are associated with earlier onset and severe phenotypes [27, 28], suggesting an association between high penetrance and earlier onset. Consistent with these observations, our present analyses demonstrated that the earlier-onset FMF patients had a higher frequency of MEFV exon 10 mutations with high penetrance. Interestingly, our analyses also showed that E84K in exon 1 mutations was significantly more frequent in patients with earlier onset. We confirmed these results by performing the sensitivity analysis excluding the FMF cases with MEFV exon 10 mutations.

The age at disease onset is variable in FMF. As noted in the Introduction, the survey of 470 FMF cases in the 1960s showed that approximately 90% of patients experienced their first attack before 20 years of age, and the onset of FMF at > 40 years of age was rare [5]. A survey in the 2000s, when FMF had gradually come to be recognized, showed that the proportions of FMF patients whose age at onset was over 20 years and over 40 years were 14% and 1.25%, respectively [20]. Thus, later-onset FMF has been considered rare worldwide.

In contrast, later-onset FMF is more common in Japan compared to Western countries. Two studies from Japan revealed that the mean ± SD age at onset is 24.2 ± 18.1 years [9] and 23.7 ± 13.6 years [17], respectively. The present study showed that age at onset > 20 years and > 40 years occurred in 52% (205/395) and 23% (90/395) of all FMF patients, and our results support the idea that adult-onset FMF is not rare in Japan. We speculate that the genetic characteristics of Japanese FMF patients (i.e., with a lower percentage of *MEFV* exon 10 mutations and a higher percentage of *MEFV* exon 2 mutations [9]) may explain the reason for the higher percentage of later-onset Japanese FMF.

A delay in the diagnosis of FMF often occurs, even in endemic areas [20, 22]. Because of self-limiting attacks that occur in a short period, FMF patients may not see a doctor or may not be referred to a specialized department, making it difficult to diagnose FMF correctly in the early course of the disease. Two studies from endemic areas revealed that the mean delay before diagnosis was 6.0 ± 6.6 years (adult onset) versus 12.1 ± 9.0 years (others) [20] and was 4.9 ± 5.8 years (late onset) versus 20 ± 13 years (others) [21], respectively. In line with these observations, our present findings indicate that the age at earlier disease onset caused a delay in diagnosis. This may be attributed to more attention being paid by adults to new manifestations and more effort being made to receive a final diagnosis [21, 22].

Most studies of childhood FMF demonstrated that FMF affects both sexes equally [5, 29–32]. It was also shown that the proportion of males was not significantly different between the young-onset and adult-onset groups [20]. However, other studies showed that later-onset FMF is characterized by male predominance [21, 22]. This is because women with later-onset FMF present a milder disease phenotype, resulting in a lesser likelihood of being diagnosed with FMF [21]. In the present study, although the proportion of males tended to be higher in the AOFMF group than in the YOFMF group, there was no significant difference between the two groups.

Our previous investigation demonstrated that the presence of *MEFV* exon 10 mutations was associated with typical FMF presentation and that typical FMF had a higher frequency of a family history of FMF [9]. Our present findings showed that earlier-onset FMF patients have significantly higher frequency of a family history as well as *MEFV* mutations in exon 10. We considered that high-penetrance mutations such as exon 10 increase the frequency of a family history. We also observed that the YOFMF group had a significantly higher frequency of typical FMF cases, probably because YOFMF patients more frequently have *MEFV* mutations in exon 10.

Our study showed that earlier-onset FMF patients predominantly present serositis manifestations including peritonitis and pleuritis, while later-onset FMF patients predominantly present musculoskeletal manifestations including synovitis and myalgia. Conversely, earlier studies showed that arthritis and erysipelas-like erythema are less frequent in adult-onset FMF compared to young-onset FMF [20, 22], which differs from our observations. However, these earlier studies did not perform sufficient analyses of the genetic differences with age at onset. Similar to our observations, a recent study from a Western country showed that LOFMF patients presented a high frequency of arthritis without significant difference and significantly less frequent chest pain compared to patients with a disease onset before 40 years of age [33]. It may thus be important to distinguish later-onset FMF from other inflammatory diseases such as crystalline-induced arthropathies and infectious arthritis.

Our multiple logistic regression analysis revealed that the presence of *MEFV* exon 10 mutations and earlier onset were significantly associated with serositis during attacks. Although this differs from some previous reports [20, 22], our analyses also revealed that the absence of *MEFV* exon 10 mutations, later onset, and the presence of erysipelas-like erythema were significantly associated with musculoskeletal manifestations. Collectively, our data indicate that the *MEFV* mutations in exon 10 with high penetrance are associated with both a high frequency of serositis and a low frequency of musculoskeletal manifestations. Japanese FMF patients not only have a lower percentage of *MEFV* exon 10 mutations but also a lower percentage of *MEFV* homozygous mutations associated with high penetrance [9, 17]. In addition, no Japanese FMF patients have the M694 V mutations in exon 10 mutations, which is especially high penetrance [9, 17]. The discrepancy between our findings and those of previous studies may be explained by the genetic characteristics of Japanese FMF patients, who have a lower percentage of *MEFV* mutations with high penetrance, especially in later-onset FMF. In addition, the discrepancy may be associated with racial differences including genetic characteristics other than the *MEFV* gene. This study is the first to describe the characteristics of FMF patients with adult onset and late onset in a country (Japan) other than endemic areas, suggesting different characteristics of FMF patients with later onset between endemic areas and other areas. We await the further accumulation of reports from locations other than endemic areas. Interestingly, the presence of erysipelas-like erythema was the strongest factor determining the presence of musculoskeletal manifestations. It was reported that the proportions of arthritis and erysipelas-like erythema are correlated [20, 22, 34], thus suggesting that these manifestations may develop with similar pathological conditions.

Since colchicine is primarily effective as a prophylactic treatment for FMF attacks, colchicine is recommended in all FMF patients regardless of the frequency and intensity of attacks. Later-onset FMF patients were described as having a milder form of disease and more favorable responses even to low-dose colchicine [21–23]. Most of the FMF patients in the present study had a good response to colchicine and there was no significant difference in secondary amyloidosis suggesting a severe phenotype among the three groups. There is a report showing that FMF patients with high-penetrance M694 V mutation in exon 10 needed higher-dose colchicine to achieve a good response [28], and there is also a report showing these patients have a significantly lower frequency of complete response to colchicine compared to patients with other MEFV mutations [35], suggesting that there may be an association between high-penetrance mutations and good response to colchicine. We suspect that Japanese FMF patients have good response to colchicine irrespective of age at onset and that there is no significant difference among the present three patient groups because of the higher frequency of low-penetrance mutations.

There are some study limitations to acknowledge. First, it remains questionable whether the diagnosis of FMF was correct in all of our cases. The diagnosis of FMF should be made based on clinical findings, not on the presence of *MEFV* gene mutations [36]. We also diagnosed FMF based on clinical findings in the present study. However, other hereditary autoinflammatory diseases cannot be completely ruled out. In addition, a good response to colchicine itself is one of the diagnostic criteria [7], and thus it is possible that patients with a poor response to colchicine were diagnosed as non-FMF.

Second, although we concluded an association with musculoskeletal symptoms and older-onset FMF by a sensitivity analysis after excluding patients with rheumatic disease, a few cases in later-onset FMF may have presented musculoskeletal symptoms due to the presence of subclinical rheumatic diseases. It has been reported that the *MEFV* gene mutations can be a risk for rheumatic diseases such as AOSD [37] and BD [38], and can modify clinical phenotypes of rheumatic diseases such as RA [39] and SLE [40]. In addition, it is generally known that the incidence of autoimmune diseases increases in proportion to age, and it is possible that rheumatic diseases before onset may be included in the adult-onset group or the late-onset group. Although each rheumatologist examined other overlapping rheumatic diseases at the diagnosis of FMF, there was no detailed information available on the profiles of autoantibodies.

Finally, there are no established standard criteria to evaluate the disease activity of FMF and the effectiveness of colchicine, and we were thus unable to evaluate these parameters accurately in the present study. The International Severity Score for FMF (ISSF) was recently recommended as a new criterion for evaluating the disease activity of FMF [41], and the FMF 50 score [42] is also recommended as a new criterion for evaluating the effectiveness of treatments such as colchicine and the necessity of intensive treatment. In the future, it is necessary to prospectively compare the disease activity and good response rate to colchicine of patients with young-onset, adult-onset, and late-onset FMF.

## Conclusions

This is the first study to describe the characteristics of Japanese FMF patients with adult onset and late onset. Our results indicate that the later-onset FMF patients had a lower percentage of mutations in exon 1 and exon 10 of the *MEFV* gene, and they presented a higher frequency of musculoskeletal manifestations and a lower frequency of serositis during their attacks. It is thus important to distinguish their FMF from other inflammatory diseases such as crystalline-induced arthropathies and infectious arthritis.

## Additional file

Additional file 1: **Figure S1.** Patient enrollment flow chart for the sensitivity analysis (TIFF 1521 kb)

## Abbreviations

AOFMF: Adult-onset FMF
AOSD: Adult-onset Still’s disease
BD: Behçet’s disease
CI: Confidence interval
CRP: C-reactive protein
ESR: Erythrocyte sedimentation
FMF: Familial Mediterranean fever
ISSF: International Severity Score for FMF
LOFMF: Late-onset FMF
OR: Odds ratio
PCR: Polymerase chain reaction
RA: Rheumatoid arthritis
SAA: Serum amyloid A
SLE: Systemic lupus erythematosus
SS: Sjögren’s syndrome
WBC: White blood cell count
YOFMF: Young-onset FMF
