# Supplementary figures and images for: Musculoskeletal manifestations occur predominantly in patients with later-onset familial Mediterranean fever: Data from a multicenter, prospective national cohort study in Japan
Source: Arthritis Res Ther. 2018 Nov 20;20:257. doi: 10.1186/s13075-018-1738-1 (PMC6247522; doi:10.1186/s13075-018-1738-1)

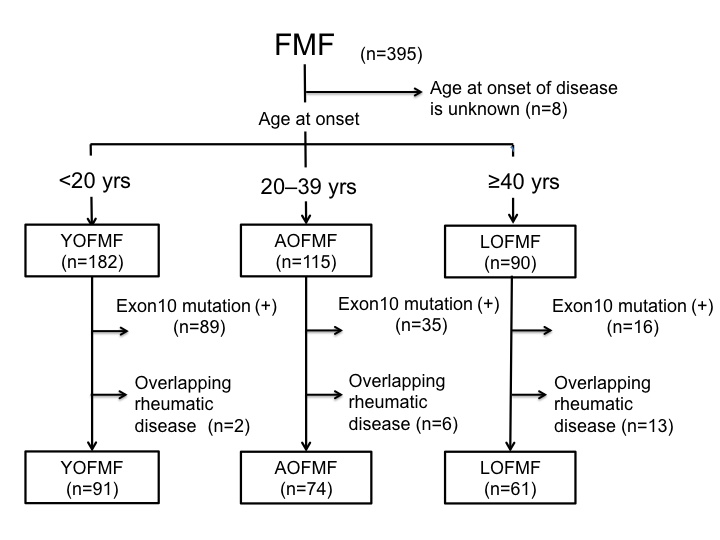

Supplement: Supplementary file 1 — Figure S1. Patient enrollment flow chart for the sensitivity analysis (TIFF 1521 kb) [file 13075_2018_1738_MOESM1_ESM.tiff]
